# Thermo-Economic Optimization of an Idealized Solar Tower Power Plant Combined with MED System

**DOI:** 10.3390/e20110822

**Published:** 2018-10-26

**Authors:** Yanjie Zheng, Yunsheng Zhao, Shen Liang, Hongfei Zheng

**Affiliations:** School of Mechanical and Vehicular Engineering, Beijing Institute of Technology, Beijing 100081, China

**Keywords:** thermo-economic optimization, solar tower power plant, endoreversible engine, dual-purpose power plant

## Abstract

Based on the reversible heat engine model, theoretical analysis is carried out for economic performance of a solar tower power plant (STPP) combined with multi-effect desalination (MED). Taking total revenue of the output power and the fresh water yield per unit investment cost as the economic objective function, the most economical working condition of the system is given by analyzing the influence of the system investment composition, the receiver operating temperature, the concentration ratio, the efficiency of the endoreversible heat engine, and the relative water price on the economic parameters of the system. The variation curves of the economic objective function are given out when the main parameter is changed. The results show that the ratio of water price to electricity price, or relative price index, has a significant impact on system economy. When the water price is relatively low, with the effect numbers of the desalination system increasing, and the economic efficiency of the overall system worsens. Only when the price of fresh water rises to a certain value does it make sense to increase the effect. Additionally, the threshold of the fresh water price to the electricity price ratio is 0.22. Under the conditions of the current price index and the heliostat (or reflector), the cost ratio and the system economy can be maximized by selecting the optimum receiver temperature, the endoreversible heat engine efficiency, and the optimum concentration ratio. Given the receiver surface temperature and the endoreversible heat engine efficiency, increasing the system concentration ratio of the heliostat will be in favor of the system economy.

## 1. Introduction

The solar tower power plant (STPP) is considered one of the most promising solar power generation technologies. There are already dozens of STPPs in operation or construction in the world. The largest of them can reach up to 100 MW [1]. In 2050, the installed capacity of 120 GW, 405 GW, or even 1000 GW could be reached globally, in which the concentrated solar power (CSP) will meet 13–15% of global electricity demand [2]. STPP technology offers the greatest potential for high efficiency. However, at the current stage, it is not economically comparable to conventional power plants. Further efforts should be made to increase the efficiency and reduce the cost [3]. Huge amounts of waste heat are released during STPP operation. Utilizing the waste heat to realize seawater desalination is an effective method to enhance the system economy. As the power plant itself also demands a large amount of fresh water, seawater desalination using its own waste heat can address the issue of water. Therefore, it is of great significance to analyze the process and the economy of the STPP combined with a multi-effect desalination (MED) system.

In general, solar collecting systems and heat engine power generation systems need to be well matched to produce the best economic benefits. How to achieve the best match between the two has always been a problem for designers of solar power plants. To this end, many researchers gave theoretical economic analyses for several kinds of solar power systems, and proposed optimum operating conditions and construction parameters. For example, De Vos [4] proposed an endoreversible thermoeconomics model for general practical engines. Sahin et al. [5] especially gave an analysis for a solar-driven heat engine. Barranco et al. [6] proposed the thermoeconomic optimum operation conditions of a solar-driven heat engine. Ust [7] combined heat transfer on the thermoeconomic performance of irreversible solar-driven heat engines and gave the variation curves of the thermoeconomic efficiency with the thermal resistance. Because construction investment of an STPP is huge, any carelessness can greatly affect the economic benefits of the system. Thus, it is of utmost importance to give economic analysis for an STPP system. For this reason, many researchers have explored the economics of STPPs. For instance, Spelling et al. [8] analyzed and discussed the thermoeconomic problem for a combined-cycle tower power system, focusing on the impact of tower system energy storage on the economics of the system. Yang [9] and Behar [10] compared and analyzed the economics of STPPs in actual operation and presented the optimum operating conditions for specific power plants. Zheng et al. [11] proposed an ideal model for STPPs, and analyzed its performance based on the endoreversible engine model, to give the optimum operating condition, including the optimum receiver temperature and the optimum efficiency of an endoreversible heat engine. Ye et al. [12] discussed the economics for a power plant based on an ideal STPP model, to calculate the most economical operating condition.

On the other hand, the combination of STPPs and seawater desalination systems is a current trend, because solar power plants themselves also demand large amounts of fresh water. Hence, many scholars have conducted theoretical explorations in this area. Trieb et al. [13] first presented a research work about the combination between desalination and CSP, explaining the option of CSP for seawater desalination, either using electricity or steam, and proving with economic data the potential of CSP+D (concentrated solar power with desalination) in the MENA (Middle East and North Africa) region. Olwig et al. [14] further performed a techno-economic analysis of a seawater desalination plant combined with a CSP. The economic indicators of the system operation under specific conditions were given. Palenzuela [15] conducted a techno-economic analysis to different kinds of large-scale solar desalination by combination with CSP and proposed several kinds of solutions for solar power plants combined with seawater desalination. Ortega-Delgado [16] undertook a thermo-economic comparison for a seawater desalination process in a concentrating solar power plant of 5 MWe. Specifically, he analyzed the coupling performance between MED and RO (reverse osmosis) units and PT-STPPs (Parabolic Trough-Solar Thermal Power Plants) with direct steam generation. Frantz and Seifert [17] gave the thermal analysis of a multi-effect distillation plant powered by a solar tower plant and simulated the relationship between electricity production and fresh water production. All these studies provide a good basis for further analysis of the economic analysis on a solar power plant combined with a seawater desalination system.

In this paper, the economics of a STPP combined with a MED system will be discussed from the perspective of the combination of thermodynamics and economics. This paper will take the maximum revenue of the sum of the power generation and fresh water production per unit investment cost as the objective function, to find the optimal economic working conditions. In this paper, an optimized economic model is proposed to obtain the relationship between the power generation and fresh water production, and the important parameters, such as the optimal concentration ratio, the receiver operating temperature, the component investment ratio, and the endoreversible heat engine efficiency.

## 2. Theoretical Model of Solar Power Tower Plant Combined with MED System

**Figure 1:**
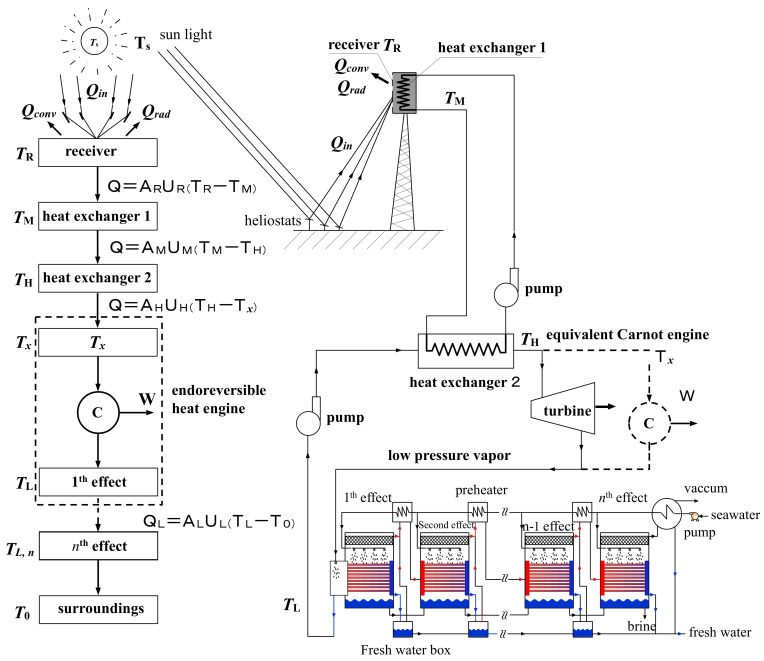
An idealized solar tower power plant combined with a multi-effect desalination (MED) system.

The composition and control of the STPP system are quite complicated. When the system is under stable operating conditions, and accurately tracks the sun, a simplified system configuration and the thermodynamic processes of the components is shown in Figure 1. From Figure 1, it can be seen that the system considers the convection and radiation losses of the receiving tower. In the thermodynamic process, the heat transfer resistance between components is also taken into account.

In the system shown in Figure 1, it is assumed that the solar irradiance input to the heliostat field of the STPP system is denoted by $I_{s}$, the concentration ratio of the system is denoted by *C*, the light entrance area of the receiver is $A_{R}$, the absorptivity of the receiver is $\alpha_{R}$, the surface operating temperature of the receiver is $T_{R}$, the ambient temperature is $T_{0}$, and the radiation heat transfer from the environment to the receiver surface is negligible. The useful heat that can be obtained by the receiver can be written as [11]:

(1) $$Q=A_{R}\left[C\alpha_{R}I_{s}-h_{c}\left(T_{R}-T_{0}\right)-\varepsilon_{R}\sigma T_{R}^{4}\right]$$

where $\varepsilon_{R}$ is emissivity of the receiver surface, $h_{c}$ is convective heat transfer coefficient between receiver and environment, and σ is the Stefan–Boltzmann constant. Reference [11] gives a thermodynamic analysis for an idealized solar tower power plant, and proposes a relation between power output and system operating or structural parameters:

(2) $$W=Q\eta =Q\left(1-\frac{T_{o}}{T_{R}-QR}\right)$$

where Q is the sum of heat gained by the system. η is the endoreversible heat engine efficiency. *R* is the total thermal resistance of the system, $R=R_{H}+R_{L}$, where $R_{H}$ is the total thermal resistance of the front end of the power system, and $R_{L}$ is total thermal resistance of the rear end of the power system, including the thermal resistance of the exchangers of the desalination system.

## 3. Thermo-Economic Optimization of Solar Tower Power Plant Combined with MED System

### 3.1. Thermo-Economic Model

Economic analysis is utilized to analyze input and output for a specific system. The input of the system refers to the sum of the construction costs and the maintenance costs. The output refers to the sum of the gains from the power output of the system and the gains from the fresh water production. Therefore, the economic efficiency of the system can be described by the ratio of output to input.

Assuming that the total amount of electricity generated during the lifetime of the solar tower thermal power plant is denoted by *W*, total amount of fresh water production is denoted by $m_{e}$, and overall investment of the system is denoted by *M*, then the investment benefit of the power plant can be expressed as:

(3) $$F=\frac{W\cdot \beta +m_{e}\cdot \gamma }{M}\;$$

where β is electricity price, RMB/kWh (RMB approximately equals 0.15 US dollars), γ is fresh water price, RMB/kg. These prices have considered the operational cost of the system. The smaller the F, the worse the system economy. Thus, the maximum value of F should be pursued.

In Figure 1, the waste heat (without considering any other use value) released from the condenser of the steam power system is denoted by $Q_{L}$. In this paper, a set of low-temperature multi-effect seawater desalination system is adopted to substitute the condenser to achieve the dual-purpose power plant. Based on the endoreversible heat engine model in Reference [11], the heat released from the system is calculated as:

(4) $$Q_{L}=Q\left(1-\eta \right)=\left(\frac{1}{\eta }-1\right)\cdot W.$$

For an ideal low-temperature MED system, under ideal conditions, the heat obtained for each effect is approximately equal, and the heat transfer area per effect is also nearly the same. Assuming there are n effects, then, in the heat release process of the steam power cycle, that is, the seawater desalination process, the total thermal resistance is:

(5) $$R=\frac{1}{A_{1}U_{1}}+\frac{1}{A_{2}U_{2}}+\cdots +\frac{1}{A_{n}U_{n}}=\frac{n}{A_{L}U_{L}}.$$

In Equation (5), A represents heat exchange area, and U represents heat transfer coefficient.

If the sensible heat consumption and the heat loss of seawater are not taken into account, the relationship between water production and $Q_{L}$ is:

(6) $$m_{e}=\frac{Q_{L}}{h_{fg,T}}\cdot n.$$

Here, $h_{fg,T}$ is water latent heat of water vaporization at temperature *T*. Assuming that 100% of the heat energy is used to evaporate seawater, substituting Equations (4) and (6) into Equation (3):

(7) $$F=\frac{W\cdot \beta +\frac{Q_{L}}{h_{fg,T}}\cdot n\cdot \gamma }{M}=\frac{W}{M}\cdot \left[\beta +\frac{n\gamma }{h_{fg,T}}\left(\frac{1}{\eta }-1\right)\right].$$

On the other side, the system investment is directly proportional to the solar concentrator (heliostat) area, receiver area, and heat exchanger area, and can be written as:

(8) $$M\propto \rho CA_{R}+\delta \left(A_{R}+A_{M}+A_{H}+nA_{L}\right).$$

Here, ρ is the price of the receiver per unit area, $A_{M}$ is the heat exchange area of the heat exchanger 1, $A_{H}$ is the heat exchange area of the heat exchanger 2, and $A_{L}$ is the heat exchange area of the first effect seawater desalination. δ is the price of the exchanger per unit area.

If we only analyze the relationship between investment benefits and system parameters, substituting Equations (2) and (8) into Equation (7) yields the following relationship:

(9) $$F=\frac{Q}{\rho CA_{R}+\delta \left(A_{R}+A_{M}+A_{H}+nA_{L}\right)}\cdot \left[1-\frac{T_{o}}{T_{R}-QR}\right]\cdot \left[\beta +\frac{n\gamma }{h_{fg,T}}\left(\frac{1}{\eta }-1\right)\right].$$

Assuming that the heat exchangers have the same area and the same heat transfer coefficient, then

(10) $$R=\frac{1}{A_{H}U_{H}}+\frac{1}{A_{M}U_{M}}+\frac{1}{A_{R}U_{R}}+\frac{n}{A_{L}U_{L}}=\left(\frac{1}{A_{R}}+\frac{1}{A_{H}}+\frac{1}{A_{M}}+\frac{n}{A_{L}}\right)\frac{1}{U}=\frac{3+n}{UA_{R}}.$$

Hence,

(11) $$F=\frac{Q}{\rho CA_{R}+\delta A_{R}\left(3+n\right)}\cdot \left(1-\frac{T_{o}}{T_{R}-QR}\right)\left[\beta +\frac{n\gamma }{h_{fg,T}}\left(\frac{1}{\eta }-1\right)\right].$$

Reference [18] gives the convection heat transfer coefficient of the tower power plant receiver to the environment:

$$\;h_{c}\approx \frac{T_{R}}{60}+\frac{5}{3}.\;$$

Substituting the above equation into Equation (1), and substituting it into Equation (11) yields:

(12) $$F=\frac{C\alpha_{R}I_{s}-\left(\frac{T_{R}}{60}+\frac{5}{3}\right)\left(T_{R}-T_{0}\right)-\varepsilon_{R}\sigma T_{R}^{4}}{\rho C+\delta \left(3+n\right)}\times \{1-\frac{T_{o}}{T_{R}-\left[C\alpha_{R}I_{s}-\left(\frac{T_{R}}{60}+\frac{5}{3}\right)\left(T_{R}-T_{0}\right)-\varepsilon_{R}\sigma T_{R}^{4}\right]\frac{\left(3+n\right)}{U}}\}\cdot \left[\beta +\frac{n\gamma }{h_{fg,T}}\left(\frac{1}{\eta }-1\right)\right].$$

Since η is a function of $T_{R}$, Equation (12) can finally get the relationship between F and $T_{R}$.

On the other hand, it can be obtained from Equation (2) that

(13) $$Q=\frac{1}{R}\left(T_{R}-\frac{T_{o}}{1-\eta }\right).$$

Substitute it into Equation (11), then

(14) $$F=\frac{U\left(T_{R}-\frac{T_{o}}{1-\eta }\right)\times \eta }{\left(n+3\right)\left[\rho C+\delta \left(3+n\right)\right]}\times \left[\beta +\frac{n\gamma }{h_{fg,T}}\left(\frac{1}{\eta }-1\right)\right].$$

Define the proportional parameter x of the solar concentrator (heliostat) cost relative to the total investment costs:

$$\;x=\frac{\rho C}{\rho C+\delta \left(3+n\right)}.\;$$

Then, Equation (14) can be written as:

(15) $$F=\frac{Ux\left(T_{R}-\frac{T_{o}}{1-\eta }\right)\eta }{\left(n+3\right)\rho C}\times \left[\beta +\frac{n\gamma }{h_{fg,T}}\left(\frac{1}{\eta }-1\right)\right].$$

Let $\theta =\frac{\gamma }{\beta h_{fg,T}}$ be a relative price index of fresh water price versus electricity price, a new dimensionless economic objective function *f* can be defined as:

(16) $$f=\frac{\rho CF}{T_{o}U\beta }=\frac{x\left(T_{R}-\frac{T_{0}}{1-\eta }\right)\eta }{\left(n+3\right)T_{0}}\left[1+n\theta \left(\frac{1}{\eta }-1\right)\right].$$

Apparently, *f* can reflect economic benefit of the dual-purpose power plant system. The larger *f* is, the better the system economic benefit is. Equation (16) also shows that there are multiple factors that affect the system’s economic benefit. Hence, various parameters of the system should be reasonably selected to maximize the total economic benefits of the system.

### 3.2. Impacts of Relative Price Index and MED Effect Numbers on Economic Objective Function

Obviously, the economic benefits of a solar tower power plant system strongly depend on the price of water γ, that is, it strongly depends on the relative price index θ. Meanwhile, the number of MEDs has a strong influence on the economic objective function. To evaluate this effect, assume x=0.5, $T_{R}=900\;K$, $T_{0}=300\;K$, η=0.4, and substitute them into Equation (16):

(17) $$f=\frac{0.26667}{n+3}\times \left(1+1.5\theta n\right).$$

**Figure 2:**
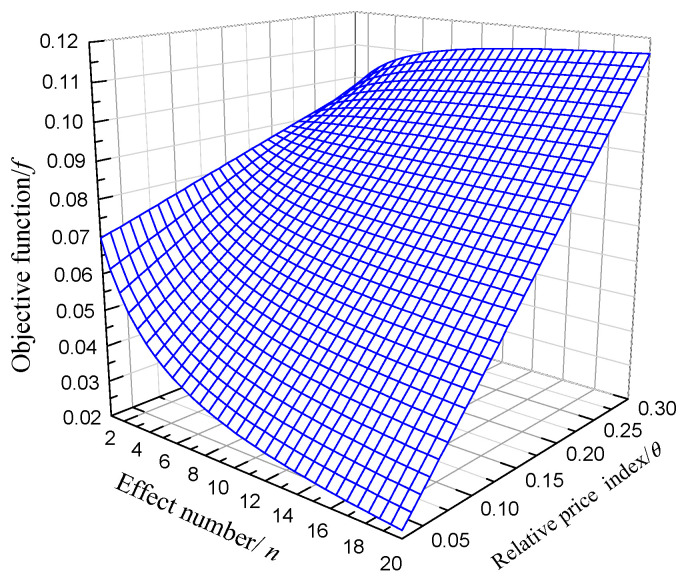
Diagram of MED effect numbers and relative price index influencing the economic objective function.

This equation shows the effect of n and θ on the economic objective function. Figure 2 shows the three-dimensional surface calculated by this equation.

**Figure 3:**
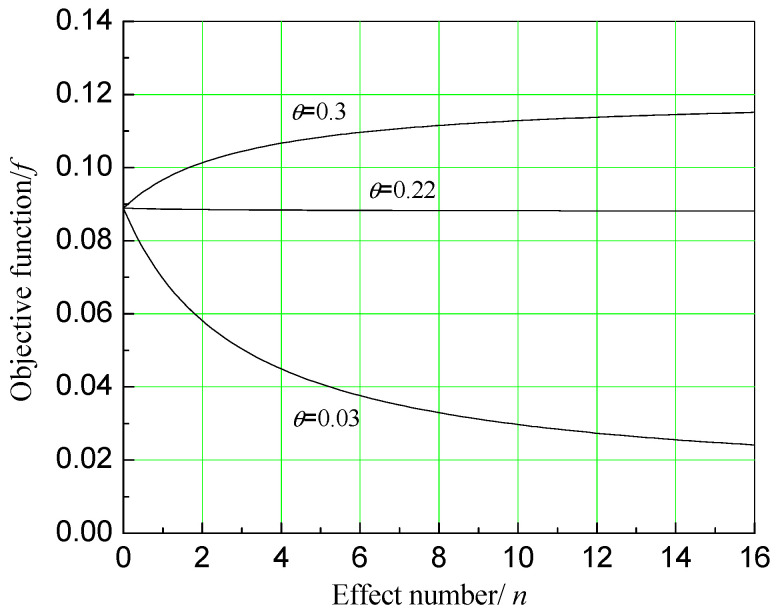
Impact of MED effect number on the economic objective function when relative price index is different.

In order to see the influence of the price index on the economic objective function more intuitively, the two-dimensional diagrams of the influence of n on the economic objective function when θ is equal to 0.03, 0.22, and 0.3 is plotted, as shown in Figure 3. Here, θ=0.03 describes the situation when water price is 10 RMB/ton and electricity price is 0.5 RMB/kWh, which is close to fresh water price and electricity price in cities of China currently.

From Figure 2 and Figure 3, it can be observed that when relative price index θ is relatively small, no matter how the number of effects n increases, economic efficiency of the system always decreases. This shows that when the fresh water price is relatively low, or when the electricity price is relatively high, the establishment of a dual-purpose power plant has no economic benefit. Only when θ≥0.22, adding a MED system will not hurt economic benefits of the total system, and be economically meaningful. When θ is larger, it is more economical to add the seawater desalination system. In water-scarce areas, the price of fresh water soars up, making dual-purpose power plants become very meaningful. The larger θ, the more economic benefit we can get by adding a desalination system. 

### 3.3. The Impact of Endoreversible Heat Engine Efficiency on Economic Objective Function

**Figure 4:**
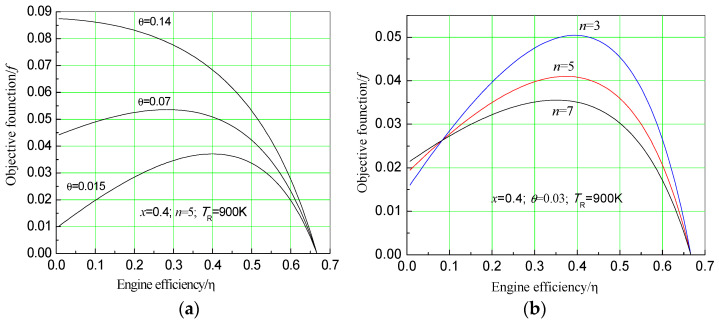
Under different engine efficiencies, impact of (**a**) different effect numbers and (**b**) different relative price indexes on the objective function.

Efficiency of the endoreversible heat engine, which can strongly affect economic objective function, is an important parameter of the system. Assuming x=0.4 (in practical system, the solar concentrator cost relative to the total investment cost is in the range of 0.35 to 0.55), $T_{R}=900\;K$, $T_{0}=300\;K$, n=3, 5, 7, θ=0.03, 0.07, 0.14, and substituting in Equation (16), the efficiency of how the endoreversible heat engine influences the objective function can be shown in Figure 4.

Based on Figure 4, when relative price index θ is small, the maximum value of the objective function decreases as number of effects of desalination system increases. But at this time, the objective function always has a maximum with the change of the heat engine efficiency. However, when θ becomes larger, objective function increases faster at less endoreversible heat engine efficiency. In addition, objective function increases more slowly at a range of higher endoreversible heat engine efficiency, causing the economic objective function to eventually change into a monotonic function with the change of the endoreversible heat engine efficiency. There will no longer be a maximum value, but the total economic benefits will still increase, as Figure 4 shows. However, it is critical to note that choice of the number of MED effects, limited by *T*_L_ and *T*_0_, cannot be increased without a limit.

### 3.4. Optimum Endoreversible Heat Engine Efficiency

From Figure 4, it can be seen that when the relative price index is small, the economic objective function has a maximum with the change of heat engine efficiency. In order to obtain the maximum value of the economic objective function and optimize the parameters, for Equation (16),

$$\;\frac{\partial f}{\partial \eta }=0\;$$

can obtain the equation with the optimum efficiency:

(18) $$\eta_{\mathrm{opt}}=1-\sqrt{\frac{T_{o}}{T_{R}\left(1-\theta n\right)}}.$$

**Figure 5:**
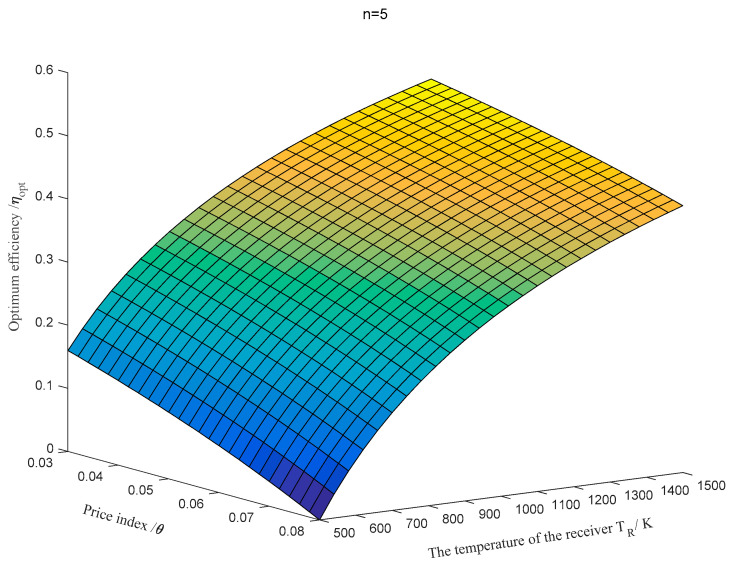
The variation of the optimum efficiency $\eta_{\mathrm{opt}}$ with $T_{R}$ and θ when *n* = 5.

Apparently, the optimum efficiency is related to receiver temperature $T_{R}$, the relative price index θ, and number of effects n of the desalination system. When n is taken as 5, the variation of $\eta_{\mathrm{opt}}$ with $T_{R}$ and θ is shown in Figure 5. It can be found that when $T_{R}$ = 1500 K and θ=0.03, the optimum efficiency is maximum, namely $\eta_{\mathrm{opt}}=0.5149$; when $T_{R}$ = 500 K and *θ* = 0.08, the optimum efficiency is maximum, namely $\eta_{\mathrm{opt}}$ = 0. This means that there is neither the optimum engine efficiency for the objective function nor the work output.

Substituting Equation (18) into Equation (16), the maximum economic objective function can be written as:

(19) $$f_{\max}=\frac{x}{\left(n+3\right)T_{0}}\left[T_{R}-\sqrt{T_{R}T_{0}\left(1-\theta n\right)}\right]\left[1-\sqrt{\frac{T_{0}\left(1-\theta n\right)}{T_{R}}}\right].$$

**Figure 6:**
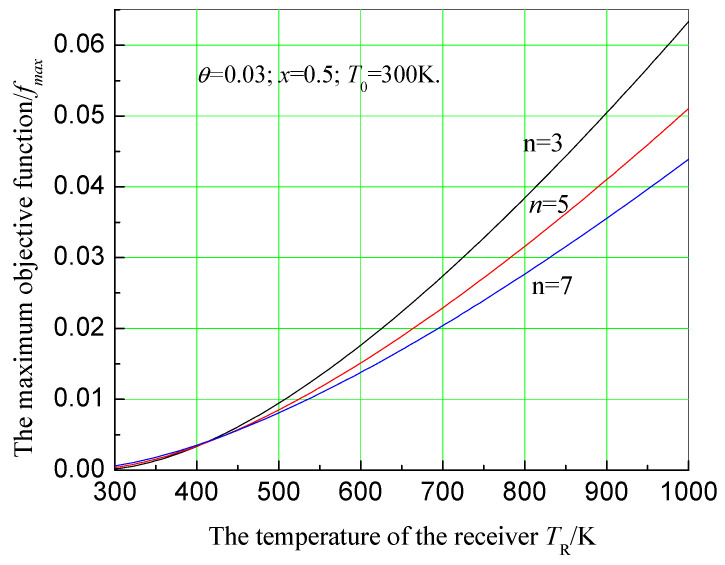
Effect of receiver temperature on maximum objective function.

Equation (19) shows that there are several parameters that could affect the maximum objective function. When *n*, θ, x, and $T_{0}$ are given, the effect of $T_{R}$ on the maximum objective function can be obtained. Let $n=3,\;5,\;7;\;\theta =0.03;\;x=0.5;\;T_{0}=300\;K$, to gain the effect of $T_{R}$ on the maximum economic objective function, as shown in Figure 6. In this figure, maximum objective function increases with the receiver temperature. However, it decreases as number of effects of the MED system increases. 

Therefore, choosing to operate at a higher receiver temperature will benefit the system economics. The number of effects of the MED system should be reasonably selected. When the relative price index is low, the number of effects should not be excessive.

### 3.5. Effect of Receiver Temperature on the System Economy

Operating temperature of the receiver surface is also an important parameter that will have an impact on the system economy. To figure out the effect of $T_{R}$ on f, transform Equation (2) into

(20) $$\eta =\left(1-\frac{T_{o}}{T_{R}-QR}\right).$$

Then, substitute Equation (20) into Equation (16):

(21) $$f=\frac{x}{\left(n+3\right)T_{o}}RQ\left[\left(1-\theta n\right)\left(1-\frac{T_{o}}{T_{R}-QR}\right)+\theta n\right].$$

Additionally, substitute

$$\;R=\frac{3+n}{UA_{R}}\;$$

$$\;Q=A_{R}\left[C\alpha_{R}I_{R}-h_{c}\left(T_{R}-T_{o}\right)-\varepsilon_{R}\sigma T_{R}^{4}\right]\;$$

into Equation (21). With $h_{c}=\left(\frac{T_{R}}{60}+\frac{5}{3}\right)$, we can get:

(22) $$f=\frac{x}{UT_{0}}\times \left[C\alpha_{R}I_{R}-\left(\frac{T_{R}}{60}+\frac{5}{3}\right)\left(T_{R}-T_{0}\right)-\varepsilon_{R}\sigma T_{R}^{4}\right]\times \{\left(1-\theta n\right)\left[1-\frac{T_{o}}{T_{R}-\left[C\alpha_{R}I_{R}-\left(\frac{T_{R}}{60}+\frac{5}{3}\right)\left(T_{R}-T_{0}\right)-\varepsilon_{R}\sigma T_{R}^{4}\right]\frac{3+n}{U}}\right]+\theta n\}.$$

**Figure 7:**
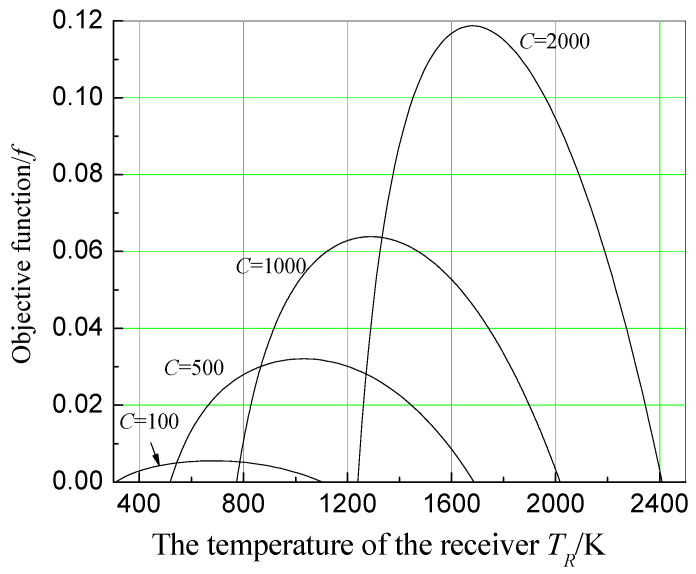
Effect of receiver temperature on economic objective function under different concentration ratios.

Assuming $\alpha_{R}=\varepsilon_{R}=1$, $I_{R}=1000\;W/m^{2}$, *U* = 15,000 $W/m^{2}\cdot K$, x=0.5, $T_{0}=300\;K$, θ=0.03, and n=5, the effect of solar receiver temperature on the objective function is calculated and plotted as shown in Figure 7 under different concentration ratio conditions. It can be seen from Figure 6 that when θ=0.03, there is always an optimum receiver temperature that makes the objective function maximum under different concentration ratio conditions. It indicates that selecting the optimum receiver temperature under different concentrating conditions is very important.

**Figure 8:**
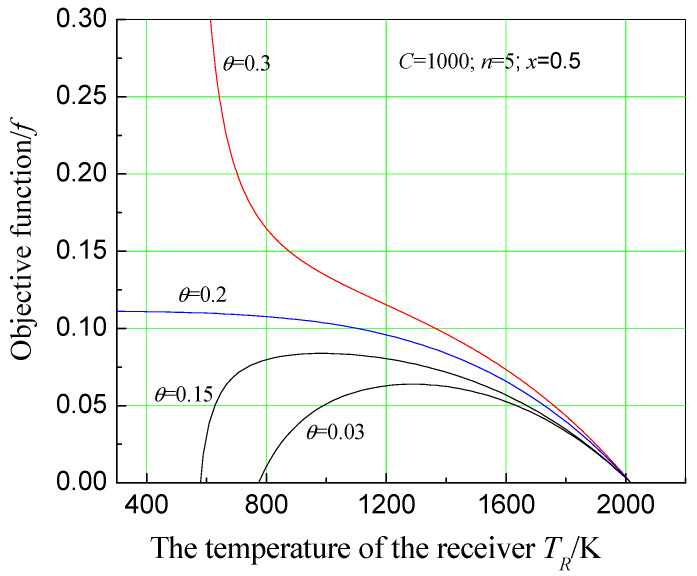
Effect of receiver temperature on economic objective function under different relative price indexes.

In addition, Figure 8 shows the effect of receiver temperature on economic objective function when the concentration ratio is 1000, and number of effects of the MED system is 5. As can be seen from Figure 8, when θ is relatively small, f has a maximum value with the change of $T_{R}$. As θ increases, maximum value of *f* increases. However, the receiver temperature corresponding to it becomes lower, resulting in *f* having no maximum when θ increases to a certain value. Understanding this trend will be of great significance to the design and implementation of a solar dual-purpose power plant.

### 3.6. Effect of Concentration Ratio on System Economics

**Figure 9:**
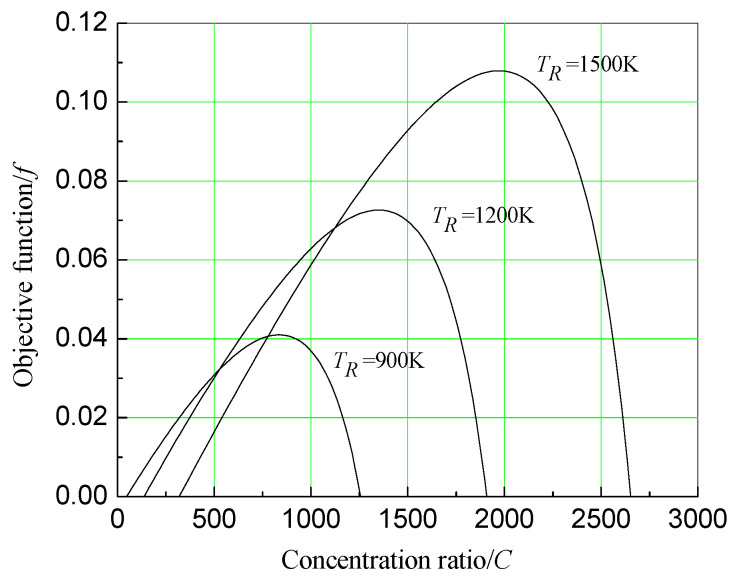
Effect of concentration ratio on economic objective function under different receiver temperatures.

**Figure 10:**
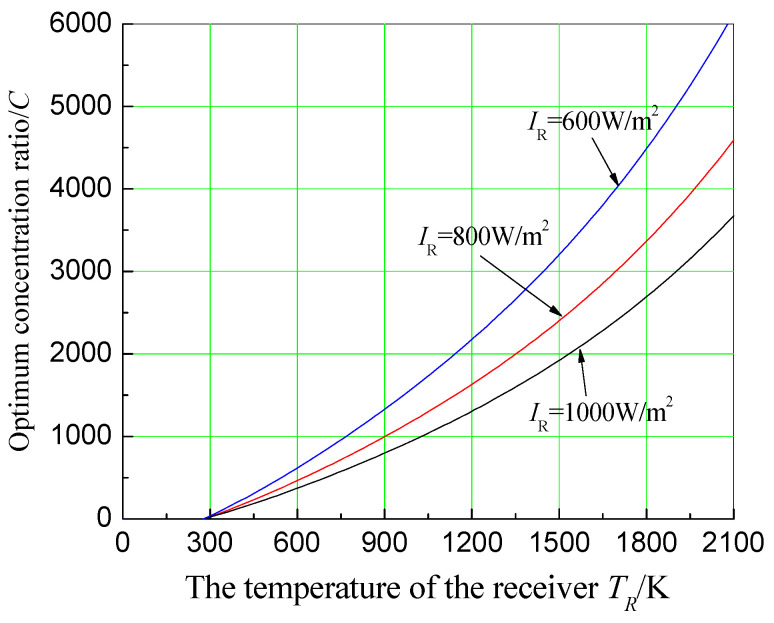
Effect of receiver operating temperature on optimum concentration ratio under different solar radiations.

It is important to select a concentration ratio for the system with a given operating temperature of the receiver. According to Equation (22), assuming $\alpha_{R}=\varepsilon_{R}=1$, $T_{o}=300\;K$, $I_{R}=1000\;W/m^{2}$, *U* = 15,000 $W/m^{2}\cdot K$, x=0.5, $T_{R}=900,\;1200\;K$ and 1500 K, θ=0.03 and n=5, the effect of a concentration ratio on system economics under different receiver temperatures is plotted in Figure 9. It can be seen from Figure 8 that, under different receiver temperatures, there is always an optimum concentration ratio that can maximize the economic objective function. For instance, when receiver temperature is 1200 K, the optimum concentration ratio is about 1400. They are close to the current working conditions of the actual system. To obtain the relationship between the optimum concentration ratio and the receiver temperature, find $\frac{\partial f}{\partial C}=0$, then get the relationship between $T_{R}$ and C, which can be shown in Figure 10. Figure 10 shows that the higher the receiver temperature, the greater the optimum concentration ratio. However, the stronger the solar radiation is, the smaller the optimum concentration ratio is.

**Figure 11:**
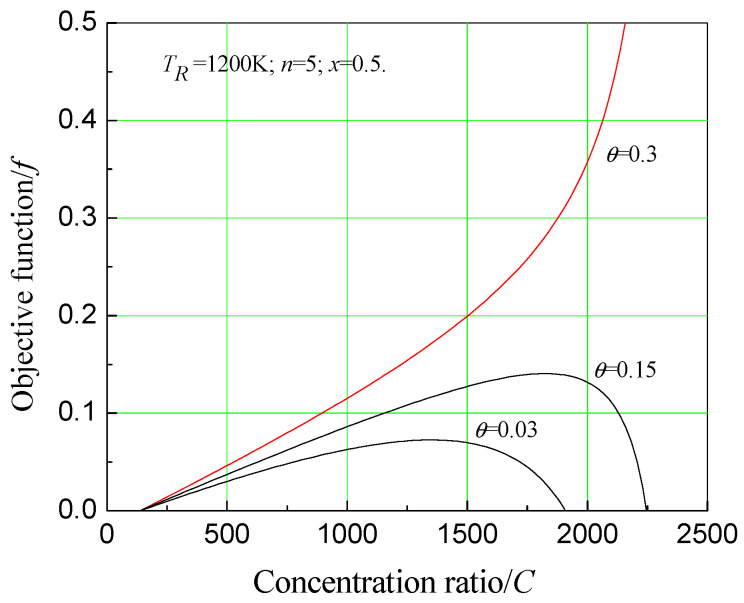
Effect of concentration ratio on economic object function under different relative price indexes.

As Figure 11 shows, when θ is relatively small, objective function exists at a maximum along with the concentration ratio variation. Additionally, the maximum objective function and the corresponding optimum concentration ratio increases as θ increases. When θ increases above a certain value, objective function becomes a monotonic function. Under this situation, a maximum value will not appear. It implies that the system has better economic performance under the condition of a larger concentration ratio.

## 4. Conclusions

After conducting comprehensive cost-benefit analysis of a STPP combined with a MED system, the objective function of the system is given. Based on the objective function, the effect of operating parameters of the system on the economic objective function is analyzed. The conclusions are described below.

(1) When water-to-electricity relative price index is small, it is meaningless to add the number of effects of the MED system. Increasing number of effects is meaningful only after water price increases above the critical value, which is θ=0.22.
(2) When the price index is relatively low, objective function can reach maximum as a function of endoreversible heat engine efficiency. However, as the price index increases, within the range of relatively low endoreversible heat engine efficiency, objective function increases faster; within the range of relatively high endoreversible heat engine efficiency, objective function increases more slowly. This makes economic objective function vary with endoreversible heat engine efficiency as a monotonic function. It means the total economic benefit will keep growing, without maximum value.
(3) When the price index is relatively low, the maximum objective function increases with receiver temperature, but decreases with the number of effects of the MED system.
(4) When the price index is relatively low, objective function can reach maximum as a function of receiver temperature. When the price index increases, maximum value of economic objective function increases, but the corresponding receiver temperature is reduced until economic objective function has no maximum value.
(5) The curve of the effect of the concentration ratio on the economic objective function is different when the relative price index is given different values. When the relative price index is small, economic objective function always has a maximum value with the change of concentration ratio. With the increase of the price index, economic objective function increases, and optimum concentration ratio increases as well. When the relative price index increases above a certain value, the economic objective function varies with concentration ratio as a monotonic increasing function, without maximum value.

## Abbreviation

$I_{s}$	solar irradiance input to the heliostat field of the solar tower
C	concentration ratio
$A_{R}$	receiver area
$T_{R}$	the surface operating temperature of the receiver
$T_{0}$	the ambient temperature
Q	the sum of heat gained by the system
$h_{c}$	convective heat transfer coefficient between receiver and environment
W	power output
R	total thermal resistance of the system
$R_{H}$	the total thermal resistance of the front end of the power system
$R_{L}$	total thermal resistance of the rare end of the power system
U	total heat transfer coefficient
F	economic efficiency of the system
$Q_{L}$	the waste heat released from the condenser
n	the effect number of MED
$h_{fg,T}$	water latent heat of water vaporization at Temperature T
$A_{M}$	the heat exchange area of the heat exchanger 1
$A_{H}$	the heat exchange area of the heat exchanger 2
$A_{L}$	the heat exchange area of the first effect seawater desalination
x	the solar concentrator (heliostat) cost relative to the total investment costs
**Greek symbols**	
θ	relative price index of fresh water price versus electricity price
f	dimensionless economic objective function
$\eta_{opt}$	optimum efficiency
$\alpha_{R}$	the absorptivity of the receiver
$\varepsilon_{R}$	emissivity of the receiver surface
σ	Stefan-Boltzmann constant
β	electricity price
γ	fresh water price
δ	the price of exchanger unit area
ρ	the price of receiver unit area
η	structural parameters
